# Ceramifiable Silicone Rubber Composites with Enhanced Self-Supporting and Ceramifiable Properties

**DOI:** 10.3390/polym14101944

**Published:** 2022-05-11

**Authors:** Dong Zhao, Lingcheng Kong, Jiaxin Wang, Guodong Jiang, Jun Zhang, Yucai Shen, Tingwei Wang

**Affiliations:** 1 College of Material Science and Engineering, Nanjing Tech University, Nanjing 211816, China; 8113557343@njtech.edu.cn (D.Z.); 202061203168@njtech.edu.cn (L.K.); m18357330035@163.com (J.W.); 202110006969@njtech.edu.cn (G.J.); zhangjun@njtech.edu.cn (J.Z.); 2 Jiangsu Collaborative Innovation Center for Advanced Inorganic Function Composites, Nanjing 211816, China

**Keywords:** silicone rubber, composites, self-supporting, ceramifiable, fire-resistant

## Abstract

Ceramifiable silicone rubber (SR) composites with excellent self-supporting properties and ceramifiable properties were prepared by incorporating silicate glass frits (SGFs) and sodium tripolyphosphate (STPP) into the SR. Ceramic residues were obtained by firing ceramifiable SR composites at 700, 850, and 1000 °C for 30 min. The bending angles of the composites were tested for evaluating the self-supporting property. To evaluate the ceramifiable properties of the ceramifiable SR composite, flexural strength, water absorption, and bulk density of its residues were tested. It was found that the addition of STPP improved the shape stability and the self-supporting property of the composites at high temperatures. The flexural strength of the ceramic residue of the composite with STPP firing above 850 °C is more than 5 MPa. X-ray diffraction (XRD) and Fourier transform infrared spectroscopy (FTIR) analysis showed that the relative content of the crystalline phase was enhanced by about 25% due to the addition of STPP. Furthermore, a possible mechanism for the formation of the crystalline phase was proposed. Scanning elector microscope (SEM) and energy dispersive spectrometry (EDS) analysis demonstrated that with the temperature increase, the inter-infiltration between these melts became easier, which implies that the bulk density of the ceramic residue was improved.

## 1. Introduction

Development and application of ceramifiable composites have grown significantly since the beginning of the 21st century due to increasing demands of public property and high-rise buildings for fire protection regulations [1,2]. Commonly, ceramifiable polymer composites are prepared by incorporating mineral fillers, flame-retardant additives, and other additives into the polymer. They have the properties of matrix materials at room temperature and could also be transformed into ceramic-like residues at high temperatures [3,4,5]. Herein, the mineral fillers are important components for ceramifiable polymer composites, which will be melted at elevated temperatures to interact with other additives.

In the past few years, in order to improve the ceramifiable properties of the ceramifiable polymer composites, various kinds of mineral fillers have been researched [6,7,8,9,10]. As an example, Shi et al. [11] incorporated MgO-Al_2_O_3_-SiO_2_ into boron phenolic resin, hence obtaining ceramifiable phenolic resin composites. The obtained results indicated that the ceramifiable boron phenolic resin composite with 40 wt.% of inorganic fillers exhibited excellent mechanical property after firing at 1200 °C; the bending strength of its residue is 10.2 MPa. Yang et al. [12] prepared a kind of ceramifiable polymer composite by blending fumed silica, calcium carbonate, and glass dust with silicone rubber, and the samples exhibited a relatively high flexural strength of 7.94 MPa at 1000 °C. Li et al. [13] exploited the combination of aluminum hypophosphite and mica for conferring excellent mechanical and ceramifiable properties to silicone rubber material. The compression strength of the residue increased by 42.5% when part of the mica was replaced by aluminum hypophosphite.

Furthermore, fire-resistant materials with excellent ceramification especially self-supporting properties were prepared by adding phosphorus-based compounds and other additives (mineral fillers, flame retardant, etc.) into various polymers including silicone rubber, epoxy resins, polystyrene, and ethylenea-vinyl acetate copolymer [14,15,16,17,18,19,20]. The conclusion is that whitened and capsulized red phosphorus (WCRP), and ammonium polyphosphate (APP) can promote the formation of a strong ceramic residue with a crystalline phase. This can be attributed to the reaction between the phosphorated compound and other additives. The formation of the crystalline phase endows the polymer base composites with a certain self-supporting property at high temperatures [17,20].

For example, Wang et al. [17] prepared a kind of ceramifiable EVA composite by adding whitened and capsulized red phosphorus (WCRP) into the EVA matrix. XRD and FTIR analysis showed that the addition of WCRP promoted the precipitation of cristobalite and the formation of phosphates crystal, which led to an improvement in the properties of the ceramic residues. However, the process of preparing WCRP is complicated and costly. Moreover, Shen et al. [20] researched the phase and microstructure evolution of silicone rubber/ammonium polyphosphate/aluminum hydroxide/mica composites during a thermal reaction. The results showed that the new crystal formed owing to thermal decomposition products of ammonium polyphosphate reacted with other fillers, which improved the self-supporting property of the composite and the flexural strength of its ceramic residues. We also prepared a kind of ceramifiable EVA/APP/SGF composite [21] and researched the effect of APP on its ceramifiable properties. The result showed that the addition of APP improved the self-supporting property of the composites at high temperatures. However, the ammonia released from the pyrolysis of ammonium polyphosphate led to a decrease in the flexural strength of the ceramic residues. Other researchers have drawn the same conclusions [22,23]. We also confirmed that the phosphorated compounds have an important positive effect on the ceramic reaction of the ceramifiable polymer composite. In addition, some people think that ammonia gas obtained from the pyrolysis of ammonium polyphosphate is toxic and an irritant [24]. Thus, it is imperative to solve these problems.

In this study, in order to solve the above problems and optimize the ceramifiable properties of the ceramifiable silicone rubber composites, sodium tripolyphosphate (STPP) was added to silicone rubber composites. The self-supporting property of silicone rubber composites was tested. The ceramifiable properties of the composite including flexural strength, water absorption, and bulk density were tested. The phase composition of the residues was characterized by FTIR and XRD. SEM and EDS analysis were used to study the microstructure and the ceramification process. These results showed that the addition of STPP avoided the release of gas and prompted the ceramification of ceramifiable silicone rubber-based composites.

## 2. Experimental

### 2.1. Materials

**Figure 1:**
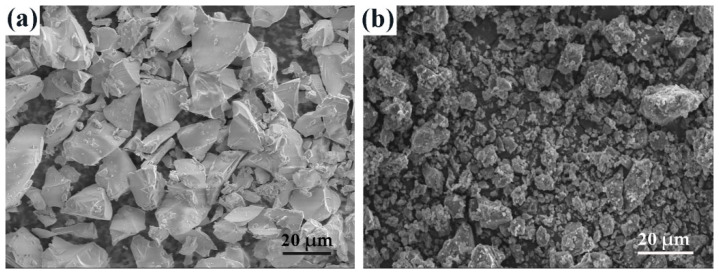
SEM images of SGFs (**a**) and STPP (**b**).

The silicone rubber (SR) (771-u) as the silicone matrix and 2,4-Dichlorobenzoyl peroxide (DCBP) as the curing agent in this work, were purchased from Jiangsu Peixing Chemical Co., Ltd. (Taizhou, Jiangsu, China). Silicate glass frits (SGFs) were provided by Jia Di Mine Product Processing Factory (Shijiazhuang, Hebei, China). SGFs are mainly composed of SiO_2_ and Na_2_O. Sodium tripolyphosphate (STPP) was supplied by Wuxi Xiongxiong Fine Chemical Technology Co., Ltd. (Wuxi, Jiangsu, China). The morphologies of SGFs and STPP were investigated using a scanning electron microscope, as is shown in Figure 1. It shows that there were no agglomerations in SGFs and STPP. SGFs and STPP were lumpy, with irregular boundaries.

### 2.2. Sample Preparation and Pyrolysis

Three sets of composites were prepared using a conventional two-roll mill. Firstly, the silicone rubber was softened at room temperature. Then, by the SGFs and STPP were mixed in until a homogeneous batch was obtained. Finally, the DCBP was added and processed until a visually satisfying dispersion was achieved. All samples were prepared through a hot-pressing process using a plate vulcanization machine. The hot-pressing technique parameters of temperature, pressure, and time were respectively 120 °C, 10 MPa, and 5 min. The formulation of all composites was described in Table 1. All samples were fired at different temperatures for 30 min in a muffle furnace.

### 2.3. Characterization

The bending angles of the ceramifiable silicone rubber composites at various high temperatures were tested as follows. Firstly, the sample was cut into cuboids, and its size was 50 × 5 × 3 mm^3^. Then the sample was put on the rectangular refractory brick, and 20% of the length of the sample extended from the brick. Finally, putting the sample along with refractory brick in the muffle furnace (KSL-1000X-M, Hefei Kejing Laboratory, Hefei, China). The bending angle of the composite was measured by Photoshop software. The smaller the bending angle is the better the self-supporting property [25].

The flexural strength of all samples fired at different temperatures for 30 min was tested by three-point bending mode using a Universal Testing Machine (LZJ-LY-98; LZJ-Technology, China) in accordance with the procedure in GB6569-86.

The crystal phase composition of samples SS, SSP-2, and SGFs powders fired at 850 °C for 30 min were characterized by an X-ray diffractometer device (Smart Lab TM 3 kW, Rigaku, Tokyo, Japan) using Cu-Kα radiation in the 2θ range of 10–80°.

FTIR spectra of residues of samples SS and SSP-2 after firing at 850 °C for 30 min were obtained by a Nicolet IS5 spectrometer (Thermo Fisher, Waltham, MA, USA) with the range of 4000–400 cm^−1^. The FTIR spectra were used to further confirm the existence of the crystals.

The water absorption and bulk density of samples after firing at 700, 850, and 1000 °C for 30 min were measured by the Archimedes principle. The water absorption of the residues was obtained via Equation (1). Ceramic residues were boiled in distilled water for 5 min, then immersed in room temperature water for 24 h.

*W =* (*M*_3_
*− M*_1_)/*M*_1_ × 100% (1)

where *W* (%) is the water absorption, *M*_1_ is the mass (g) of the dry sample, and *M*_3_ *is* the mass (g) of the wet sample. The bulk density was obtained via the Equation (2):

*D* = (*M*_1_ × *d*)/(*M*_3_ − *M*_2_) (2)

where *D* (g/cm^3^) is the bulk density. *M*_2_ and *d* are the mass (g) of the sample in water and the density of the distilled water (1 g/cm^3^), respectively.

The limited oxygen index (LOI) values were tested on an HC-2 oxygen index instrument (Nanjing Jiangning Analytical Instrument Co., Ltd., Jiangsu, Nanjing, China) with sheet dimensions of 130 × 6.5 × 3 mm^3^ according to GB/T2406 standard.

The microscopic morphology analysis of ceramic residue by a JEOL JSM 5900LV scanning elector microscope (Jeol, Tokyo, Japan). Energy dispersive spectrometry (OXFORD INCA250, Oxford, Britain) was used to analyze the elemental compositions and the elemental dispersion of the cross-section of residues.

## 3. Results and Discussion

### 3.1. Surface Morphology of All Samples Fired at Different Temperatures

**Figure 2:**
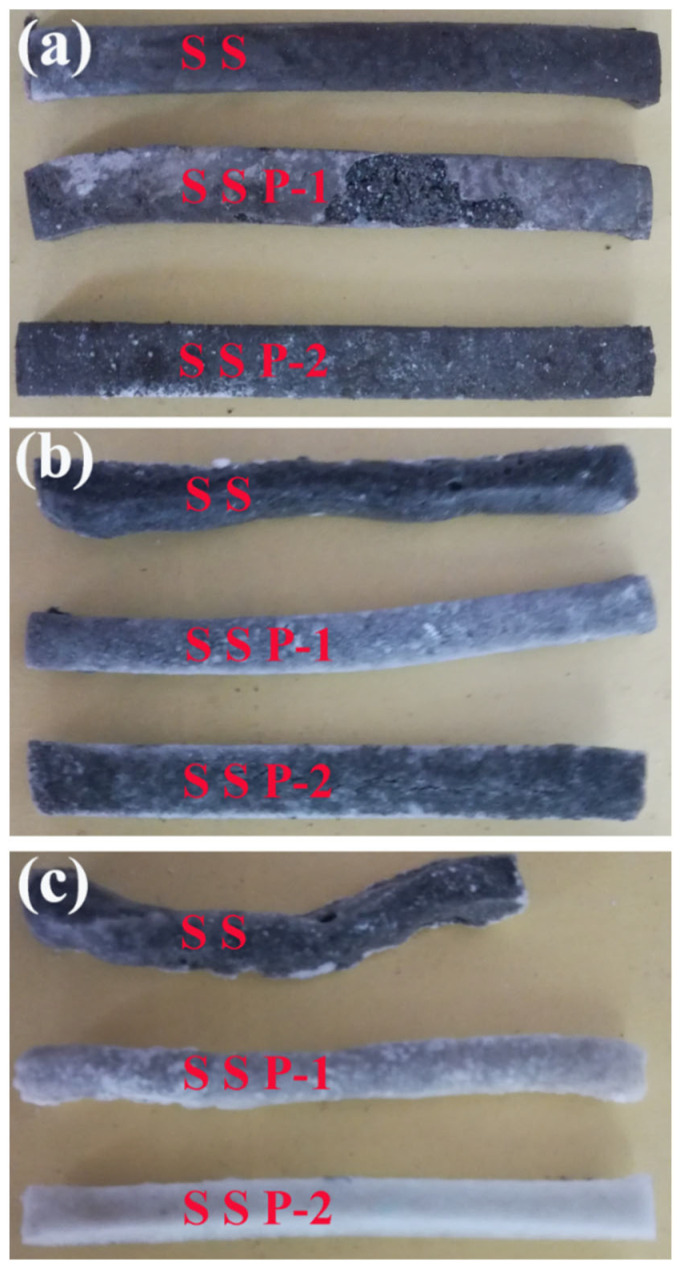
Surface morphology of all samples fired at 700 °C (**a**), 850 °C (**b**), and 1000 °C (**c**).

The influence of STPP on the morphology of the ceramic residue is shown in Figure 2. It can be seen in Figure 2a that all samples can keep their original shapes after firing at 700 °C. Figure 2b shows that with the temperature increase, the shape of sample SS began to change after firing at 850 °C, while the shapes of SSP-1 and SSP-2 kept their original shapes. Figure 2c shows that there was a significant deformation for sample SS after firing at 1000 °C. By contrast, residues of samples SSP-1 and SSP-2 essentially retained their original shapes, and no cracks and holes were observed on their surface. Therefore, the addition of STPP improves the shape stability of the composites.

### 3.2. Self-Supporting Property of Ceramifiable Silicone Rubber Composites

**Figure 3:**
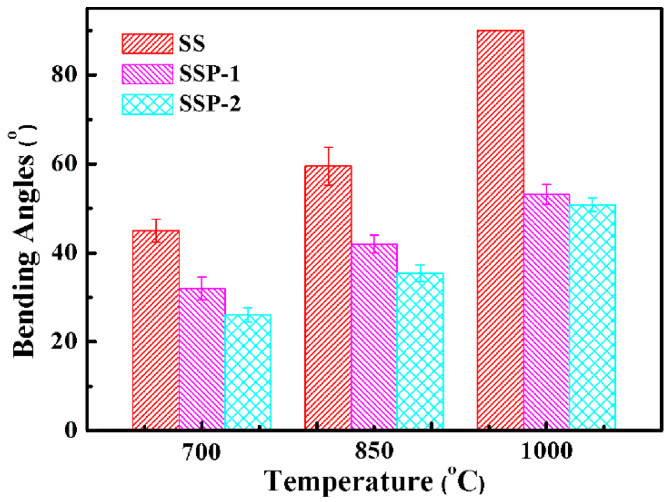
Bending angles of all samples fired at different temperatures for 30 min.

In order to evaluate the self-supporting property of the ceramifiable silicone rubber composites, bending angles of the residues were tested, as shown in Figure 3. It is obvious that with the addition of STPP, the bending angle decreased at the same firing temperature. For example, when the content of STPP increases from 0 wt% to 18 wt%, there is an obvious decrease in bending angle from 90° to 50.8 ± 2.3° for samples fired at 1000 °C. Notably, the bending angle was 90° when the sample SS was fired at 1000 °C, which means that the occurrence of melting-dropping was evident. By contrast, samples SSP-1 and SSP-2 can support their own weight to a certain extent. Therefore, it is shown that the existence of STPP can increase the crystal phase content of the residue, which improves the self-supporting property of the ceramifiable silicone rubber composite at high temperatures. Furthermore, increasing temperature results in an increase in the bending angle for each sample, which is in alignment with previous studies [17].

### 3.3. Flexural Strength of All Residues

**Figure 4:**
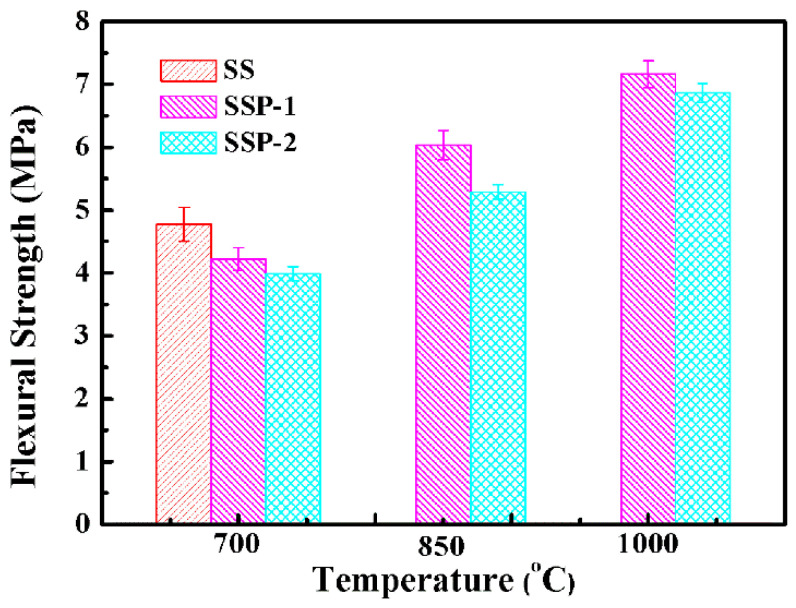
Flexural strength of all samples fired at different temperatures.

The flexural strength of sample SS fired at above 850 °C was not shown in Figure 4, because the shape of the sample SS at that condition does not meet the testing requirements. Figure 4 shows that adding STPP causes flexural strength to decrease when samples are fired at 700, 850, or 1000 °C. However, the values of flexural strength for samples SSP-1 and SSP-2 fired at high temperatures are always higher than 3 MPa, which means that they formed residues with a certain mechanical property, with increasing temperature the flexural strength increased for each sample. The flexural strength of samples SSP-1 and SSP-2 fired at 1000 °C reached 7.16 ± 0.21 MPa and 6.86 ± 0.15 MPa, respectively. Many researchers [22,23,26,27] indicate that the factors that affect the flexural strength of residues are phase composition and microstructural. Through the above analysis for the self-supporting property, we confirmed that the increase in crystal content improves the self-supporting property of the ceramifiable silicone rubber composites. Thus, its flexural strength should also be improved. As is shown in Figure 4, however, a completely opposite result is obtained, which means that the main factor affecting the flexural strength is microstructural.

### 3.4. XRD and FTIR Analysis

**Figure 5:**
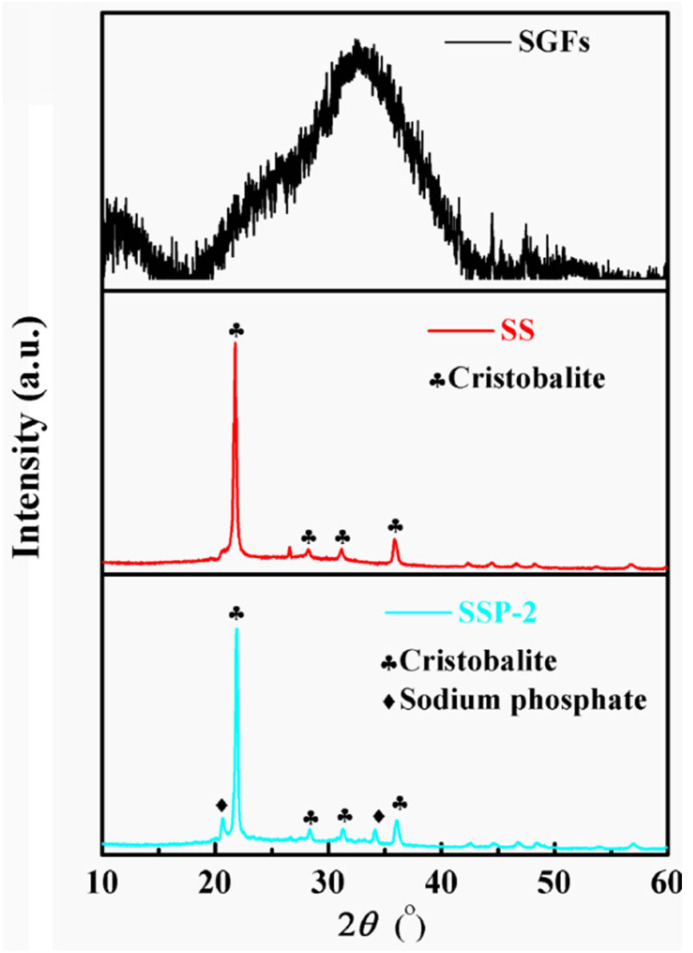
XRD patterns of SGFs, SS, and SSP-2 fired at 850 °C for 30 min.

To research the phase changes in the ceramic residues, the phase composition of the residue was characterized using XRD. Figure 5 shows that an obvious “hump” appeared at 18–42°, corresponding to the amorphous structure of SGFs after firing at 850 °C [28,29]. However, some new peaks appeared at 22.03, 28.28, 31.43, and 36.13° corresponding to cristobalite (JCPDS file no.76-936) after sample SS was fired at 850 °C [30]. XRD patterns of sample SSP-2 show two other new peaks at 20.02 and 33.25°, representing sodium phosphate (JCPDS file no.1-1103) crystals. Compared with the XRD patterns of sample SS, the crystalline peaks of cristobalite seemed to be stronger in the XRD patterns of sample SSP-2. Moreover, the quantitative calculation of crystal phase content was obtained via Jade 6.0 software [21,31], and the data was obtained by the whole pattern fitting technology. The results show that the crystal content of the sample SS is 41.47%, and the crystal content of the sample SSP-2 is 54.67%. The latter improved by about 25% over the former. According to our previous research [32], it is due to the existence of phosphorus elements, which induced the precipitation of cristobalite crystals. Therefore, the self-supporting property of silicone rubber composite was improved due to the addition of STPP.

**Figure 6:**
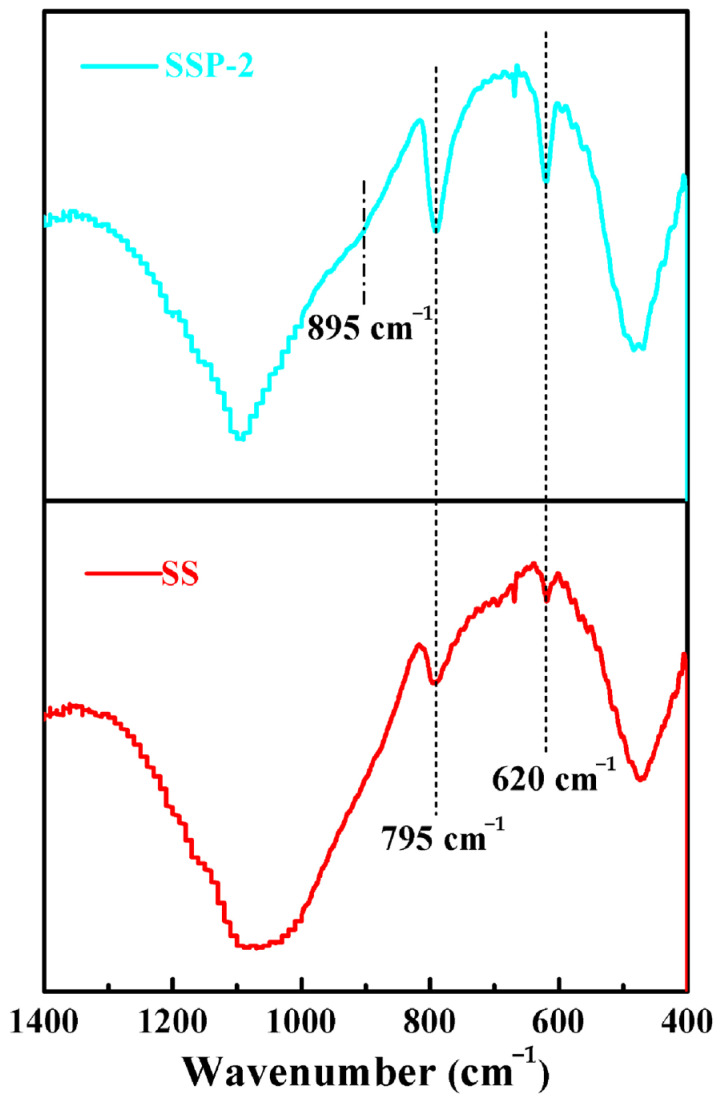
FTIR patterns of samples SS and SSP-2 fired at 850 °C for 30 min.

FTIR patterns of samples SS and SSP-2 fired at 850 °C were shown in Figure 6. Compared with the FTIR spectrum of the residue of sample SS, a new absorption band appeared at 895 cm^−1^ in the spectra of the residue of sample SSP-2, which attributed to the asymmetric stretching modes of the P-O-P bonds [19,33]. Furthermore, compared with the FTIR spectrum of the residue of sample SS, these peaks at 795 and 620 cm^−1^ became stronger in the FTIR spectrum of the residue of sample SSP-2, representing an increase in cristobalite crystals content [17]. These results coincide with the above-mentioned analysis of XRD.

### 3.5. Bulk Density and Water Absorption

**Figure 7:**
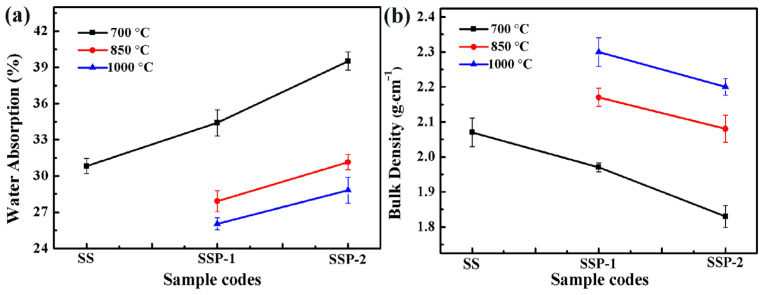
Water absorption (**a**) and bulk density (**b**) of all samples fired at 700, 850, and 1000 °C for 30 min.

Bulk density and water absorption of SS fired at 850 and 1000 °C were not measured, because the residues had tight adhesion to the refractory brick. As observed in Figure 7, for samples heated at 700, 850, or 1000 °C, the addition of STPP brings about a significant increase in the water absorption of the residues. When the content of STPP increases from 0 wt% to 18 wt%, there is an obvious increase in water absorption from 30.81 ± 0.63 to 39.52 ± 0.76% for samples fired at 700 °C. By comparison to the bulk density of all samples fired at the same temperature, the addition of STPP can further reduce the densification of the residues. When the content of STPP increases from 0 wt% to 18 wt%, there is an obvious decrease in bulk density from 2.07 ± 0.04 to 1.83 ± 0.03 g/cm^−^^1^ for samples fired at 700 °C. It is possible that the addition of STPP makes the composites burn violently. Meanwhile, the existence of crystals hinders the liquid phase to spread into the matrix.

In addition, the bulk density of all the samples is increased with the temperature increase, which coincides with the above-mentioned analysis about flexural strength. By comparison, the temperature has a greater effect on the flexural strength of residues than phase composition.

### 3.6. Limited Oxygen Index Analysis

APP, WCRP, and other phosphorus-based flame retardants were added to composites to increase the LOI, and the residues could maintain their excellent machine capability [18]. The flame retardancy of the silicone rubber composites was evaluated via LOI, and the results are shown in Table 2. As observed, STPP has a slight effect on flame retardancy for silicone rubber composites. The dosage of STPP was 0 wt.% and 18 wt.% respectively, and the limit oxygen index of the silicone rubber composites decreased from 26.5 to 25.5%. This indicates that the addition of STPP slightly enhances the flammability and reduces the thermal stability of the ceramifiable silicone rubber composites. Thus, violent burning of the composites leads to a decrease in the densification of the residues [34].

### 3.7. SEM-EDS Analysis

**Figure 8:**
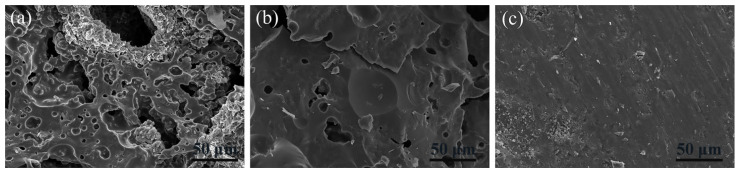
SEM photographs of the sample SSP-2 cross-sections taken under magnification of ×500; (**a**) 700 °C, (**b**) 850 °C, and (**c**) 1000 °C.

To further demonstrate the effect of temperature on the microstructure of the residues, the cross-sections of SSP-2 fired at different temperatures were observed by scanning electron microscope. Compared with Figure 1, the original morphologies of SGFs and STPP cannot be seen in Figure 8. This means that the SGFs and STPP have completely melted. Figure 8a shows that the loose and porous microstructure is formed in SSP-2 fired at 700 °C, but the continuous and less porous microstructure is formed with increased temperatures. In particular, SSP-2 fired at 1000 °C exhibits the smoothest and densest cross-section. This is supportive of SSP-2 having the lowest water absorption, highest bulk density, and highest flexural strength in these experiments.

**Figure 9:**
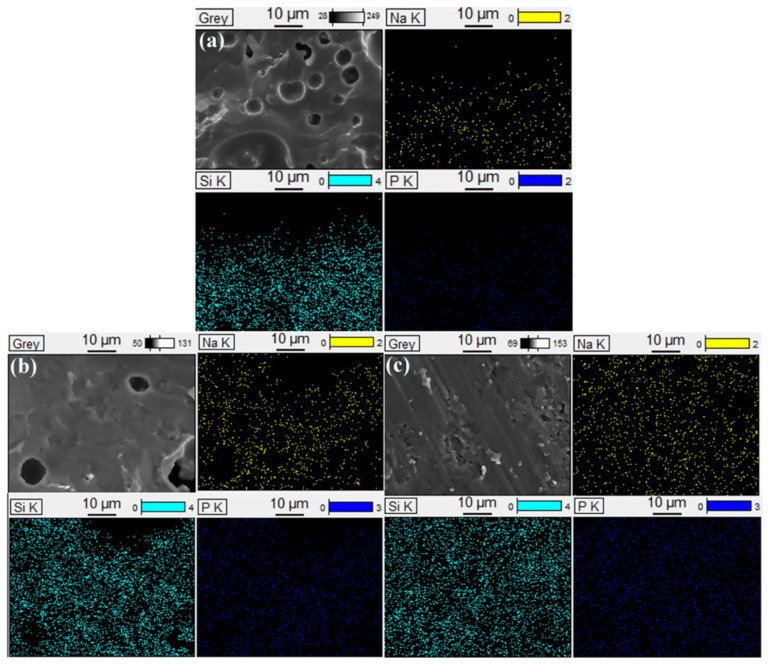
Elemental distribution mappings for the cross-sections of SSP-2 fired at different temperature; (**a**) 700 °C, (**b**) 850 °C, and (**c**) 1000 °C.

In addition, the element distribution mappings for the cross-sections of the residues are depicted in Figure 9. By comparison of the element distribution for the cross-sections of SSP-2 fired at different temperatures, the residues of SSP-2 fired at 1000 °C display a homogeneous element distribution throughout the whole area scanned. This is because a more liquid phase was formed in the residues [35]. Meanwhile, it is well known that silicon does not improve the self-supporting property of the ceramifiable silicone rubber composite. Thus, phosphates play a major role in the improvement of the self-supporting property of silicone rubber composites.

**Figure 10:**
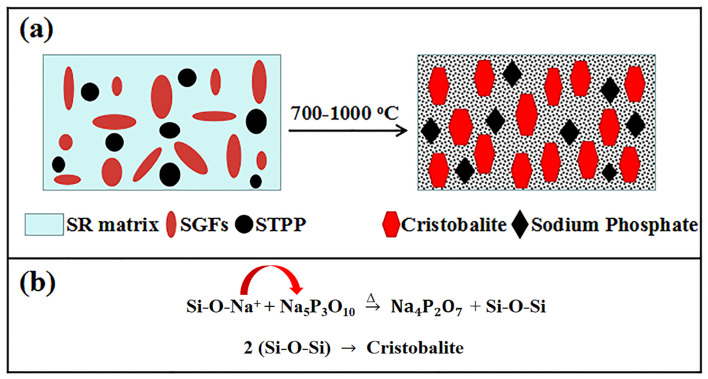
(**a**) Schematic illustrations and (**b**) chemical reaction process of the formation of cristobalite at high temperatures.

Furthermore, a possible mechanism for the formation of ceramic residue was proposed, as shown in Figure 10. The Si-rich area was formed due to sodium ions (Na^+^) from SGFs were captured by phosphates, which provided the condition for the precipitation of cristobalite [36,37], the chemical reaction process as shown in Figure 10b. Therefore, the self-supporting property of the ceramifiable silicone rubber composite was improved due to the increased crystals content.

## 4. Conclusions

In order to optimize the ceramifiable properties and self-supporting property of the silicone rubber composite, STPP and SGFs as non-toxic and without gas release fillers have been incorporated into the silicone rubber matrix. The results showed that the addition of STPP significantly improved the shape stability of the ceramifiable silicone rubber composites. Analysis of XRD and FTIR indicated that the crystal content of residues enhanced with adding STPP, which improves the self-supporting property of the ceramifiable silicone rubber composite. EDS analysis of the residue indicates that the Si-rich area was formed due to Na^+^ that was captured by P_3_O_10_^5−^, which provided the condition for the precipitation of cristobalite. The water absorption, bulk density, and SEM analysis of the residue showed a decrease in the number of pores and cracks in the residues with the increasing temperature, leading to an increase in the flexural strength. Although the STPP causes a slight decrease in the flexural strength, it still meets the requirements for industrial standards (over 3 MPa) in terms of fire-resisting cables.

## Figures and Tables

**Table 1:** Composites formulations (g).

Composition	SR	SGFs	STPP	DCBP
SS	100	100	-	2
SSP-1	100	91	9	2
SSP-2	100	82	18	2

**Table 2:** Oxygen index of ceramicable silicon rubber composites.

Composition	LOI (%)
SS	26.5 ± 0.1
SSP-1	26.0 ± 0
SSP-2	25.5 ± 0.1
